# Comprehensive genomic analysis of Oesophageal Squamous Cell Carcinoma reveals clinical relevance

**DOI:** 10.1038/s41598-017-14909-5

**Published:** 2017-11-10

**Authors:** Peina Du, Peide Huang, Xuanlin Huang, Xiangchun Li, Zhimin Feng, Fengyu Li, Shaoguang Liang, Yongmei Song, Jan Stenvang, Nils Brünner, Huanming Yang, Yunwei Ou, Qiang Gao, Lin Li

**Affiliations:** 1 BGI Genomics, BGI-Shenzhen, Shenzhen, 518083 China; 2 Section of Molecular Disease Biology, Department of Drug Design and Pharmacology, Faculty of Health and Medical Sciences, University of Copenhagen, 2200 Copenhagen N, Denmark; 3 Department of Epidemiology and Biostatistics, Tianjin Medical University Cancer Institute and Hospital, Tianjin, 300060 People’s Republic of China; 4 State Key Laboratory of Molecular Oncology, Cancer Institute and Cancer Hospital, Chinese Academy of Medical Sciences and Peking Union Medical College, Beijing, 100021 China; 5 James D. Watson Institute of Genome Sciences, Hangzhou, China; 6 Department of neurosurgery, Beijing tiantan hospital, capital medical university, Beijing, 100050 China; 7 Shanghai Clinical Center for Endocrine and Metabolic Diseases, Shanghai Key Laboratory for Endocrine Tumours, Rui-Jin Hospital, Shanghai Jiao-Tong University School of Medicine, Shanghai, 200025 China

## Abstract

Oesophageal carcinoma is the fourth leading cause of cancer-related death in China, and more than 90% of these tumours are oesophageal squamous cell carcinoma (ESCC). Although several ESCC genomic sequencing studies have identified mutated somatic genes, the number of samples in each study was relatively small, and the molecular basis of ESCC has not been fully elucidated. Here, we performed an integrated analysis of 490 tumours by combining the genomic data from 7 previous ESCC projects. We identified 18 significantly mutated genes (SMGs). *PTEN*, *DCDC1* and *CUL3* were first reported as SMGs in ESCC. Notably, the *AJUBA* mutations and mutational signature4 were significantly correlated with a poorer survival in patients with ESCC. Hierarchical clustering analysis of the copy number alteration (CNA) of cancer gene census (CGC) genes in ESCC patients revealed three subtypes, and subtype3 exhibited more CNAs and marked for worse prognosis compared with subtype2. Moreover, database annotation suggested that two significantly differential CNA genes (*PIK3CA* and *FBXW7*) between subtype3 and subtype2 may serve as therapeutic drug targets. This study has extended our knowledge of the genetic basis of ESCC and shed some light into the clinical relevance, which would help improve the therapy and prognosis of ESCC patients.

## Introduction

Oesophageal cancer is one of the most common malignant tumours in the world, and its 5-year survival rate is under 20%^1^. In China, oesophageal cancer is also one of the leading causes of cancer death, following lung, stomach and liver cancer^2^. There are approximately 478,000 newly diagnosed oesophageal cancer patients and 375,000 deaths from the disease every year in China^2^. Recently, large-scale investigations on ESCC have been performed in China, focusing on the discovery of new driver mutations that may be closely associated with the development of oesophageal cancer. Song *et al*.^3^ identified the new oncogene mutant *FAM135B*, which promoted malignant phenotypes in 17 whole genome sequencing (WGS) and 71 whole exome sequencing (WES) cases. Gao *et al*.^4^ discovered *EP300* to have tumour suppressor function in 113 WES ESCC cases and to be associated with a poor prognosis. However, due to the limited sample size, we were still unclear about the mechanisms of ESCC tumourigenesis, especially the contribution of low-frequency mutated genes^5^. Coupled with the heterogeneity of cancer mutations, a comprehensive analysis of oesophageal cancer mutation mechanisms to further the understanding of ESCC-related genes will be an important foundation for ESCC diagnosis and treatment. This study combined the genomic data obtained in seven previously published studies (Supplementary Tables 1 and 2)^3,4,6–10^ on squamous cell carcinoma to discover genes that are associated with prognosis.

## Results

### Somatic mutations in ESCC

We identified a total of 52,964 nonsilent mutations and 16,204 silent mutations in ESCC coding regions, with a median of 97 nonsilent mutations per tumour (Supplementary Tables 3–4). We then compared the nonsilent mutations of ESCC to EAC and other cancer types. The somatic mutations were highly variable between or within different cancer classes (Supplementary Fig. 1); ESCC displays fewer nonsilent mutations per tumour than EAC (median, ESCC: 97; EAC: 117.5) and a higher number than other cancers immediately below lung cancer and melanoma.

### Deciphering the mutational Signatures in ESCC

**Figure 1:**
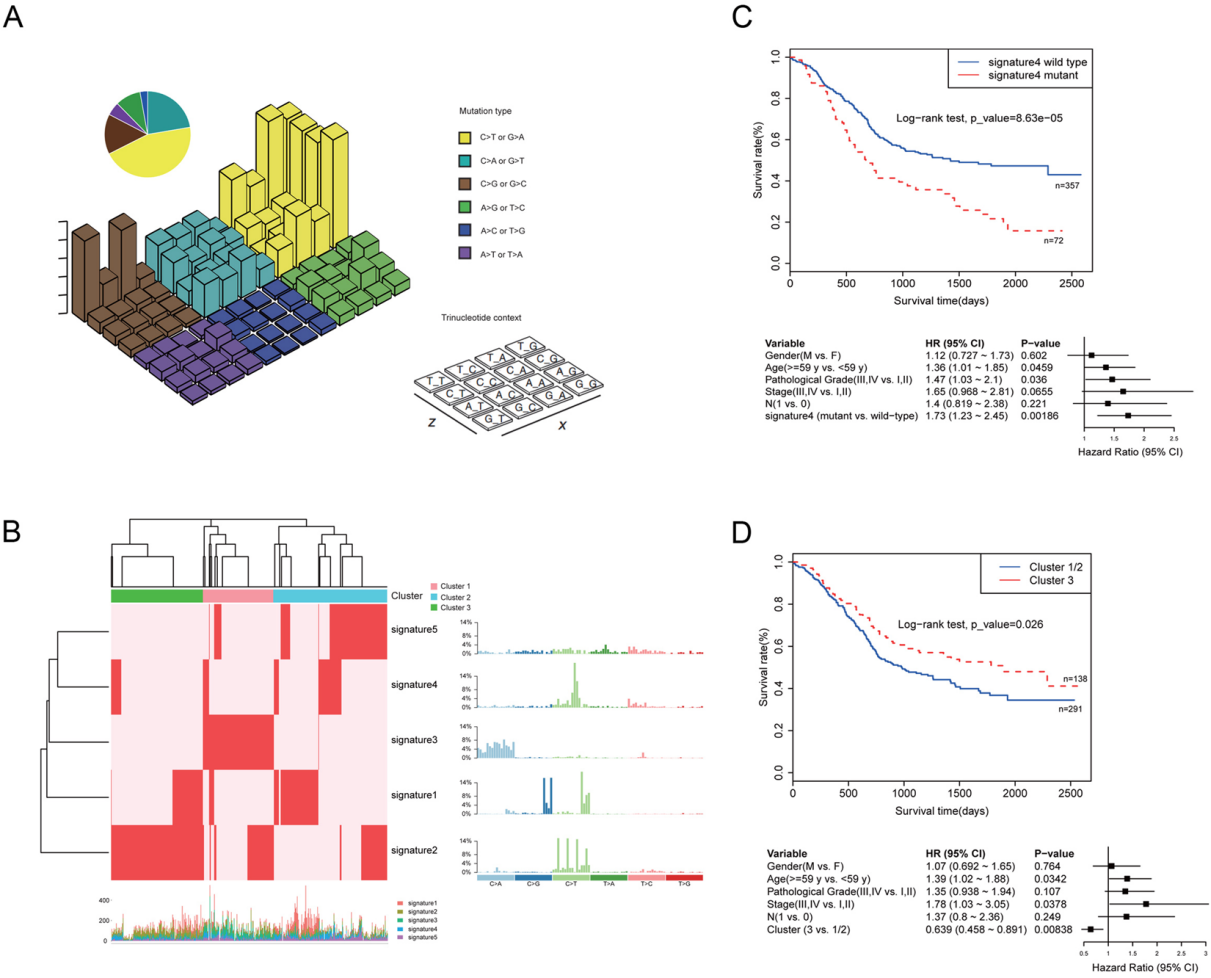
Mutational signature analysis of ESCC. (**A**) Lego plots of mutational frequencies in the coding regions in ESCC specimens. Base substitutions were classified into six subtypes and each category was represented by different colours. Pie charts represent the distribution of the six subtypes. Base substitutions were further divided into 96 possible mutation types according to the flanking nucleotides surrounding the mutated base. (**B**) Heatmap for mutational signatures using sample exposures to one signature identified in ESCC specimens by the NMF method. Each column represents one individual. Each row represents one signature. (**C**,**D**) Top: Kaplan-Meier survival curves for signature4 and cluster3 were significantly associated with patient survival, *p* < 0.1 was considered statistically significant. Bottom: Cox proportional hazards model for patients, *p* < 0.05 was considered statistically significant.

Consistent with previous studies of ESCC, the mutational spectrum showed that C:G > T:A transition was the predominant type, followed by C:G > A:T and C:G > G:C transversions (Fig. 1A, Supplementary Table 5). To further understand the process of mutation in ESCC, a non-negative matrix-factorization method was applied to decipher mutational signatures from 490 ESCC tumours, and 5 mutational signatures were generated (Fig. 1B). Signature1 was characterized primarily by C > T and C > G mutations at TpCpN trinucleotides, and has been confirmed to be associated with the APOBEC family of cytidine deaminases, which played an important role in the deaminase activity of single-stranded DNA (ssDNA)^11,12^. Signature2 was characterized by C > T mutations at NpCpG trinucleotides. This mutational process has been detected in almost all previous studies of oesophageal cancer^13^ and is related to the spontaneous deamination of 5-methyl-cytosine. Signature3, which is characterized by C > A mutations, was likely caused by tobacco mutagens^14^ and has been observed in many human cancers, including head and neck cancer, liver cancer, lung cancer, and oesophageal cancer^14^. Signature3 was observed in 127 patients, of whom 80 were smokers, accounting for 63% of these cases. Signature4 was characterized mainly by C > T mutations and was associated with defective DNA mismatch repair. Patients with signature4 exhibited poor survival (Fig. 1C). Signature5 has been found in oesophageal cancer^7^, but the aetiology of this process remains unknown. The comprehensive analysis of larger sample set enabled us to identify more comprehensive mutational signatures of ESCC and analyse the different mechanisms of carcinogenesis. Hierarchical clustering was performed based on the enrichment of specific mutational signatures, and 3 clusters were identified. Cluster1 was dominated by signature3, Cluster2 was dominated by signatures 1 and 5, and Cluster3 was dominated by signature2. The three groups were associated with different survival times. Cluster3 exhibited a better prognosis compared with patients in clusters 1 and 2 by Kaplan-Meier analysis (*p* = *0*.*026*, log-rank test) (Fig. 1D).

### Significantly mutated genes in ESCC

**Figure 2:**
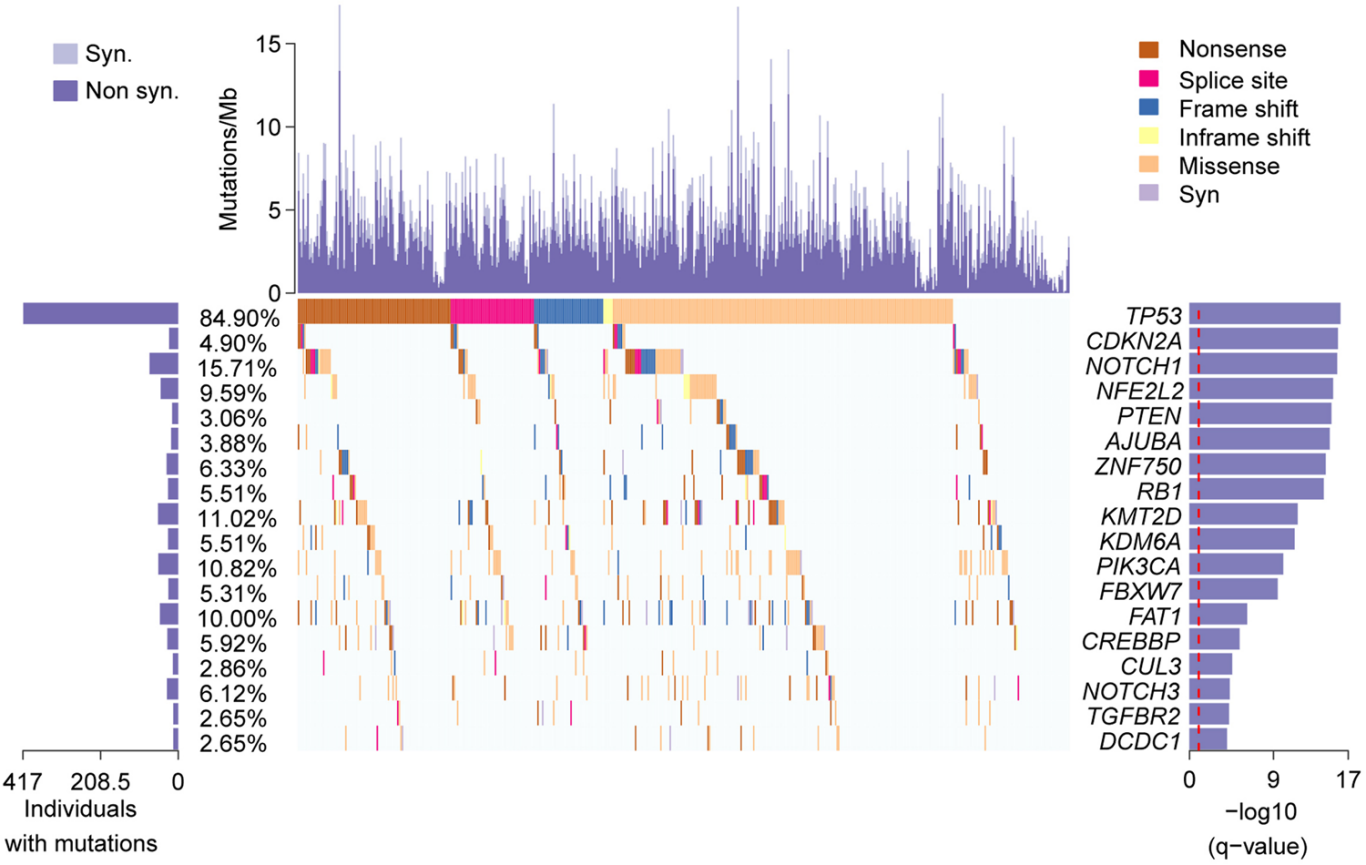
Significantly mutated genes in ESCC specimens. Top: number of synonymous and nonsynonymous mutations. Middle: significantly mutated genes coloured by mutation types. Left: Nonsilent mutation frequency of each gene. Right: significantly mutated genes ranked by q-value according to MutSigCV analysis.

**Figure 3:**
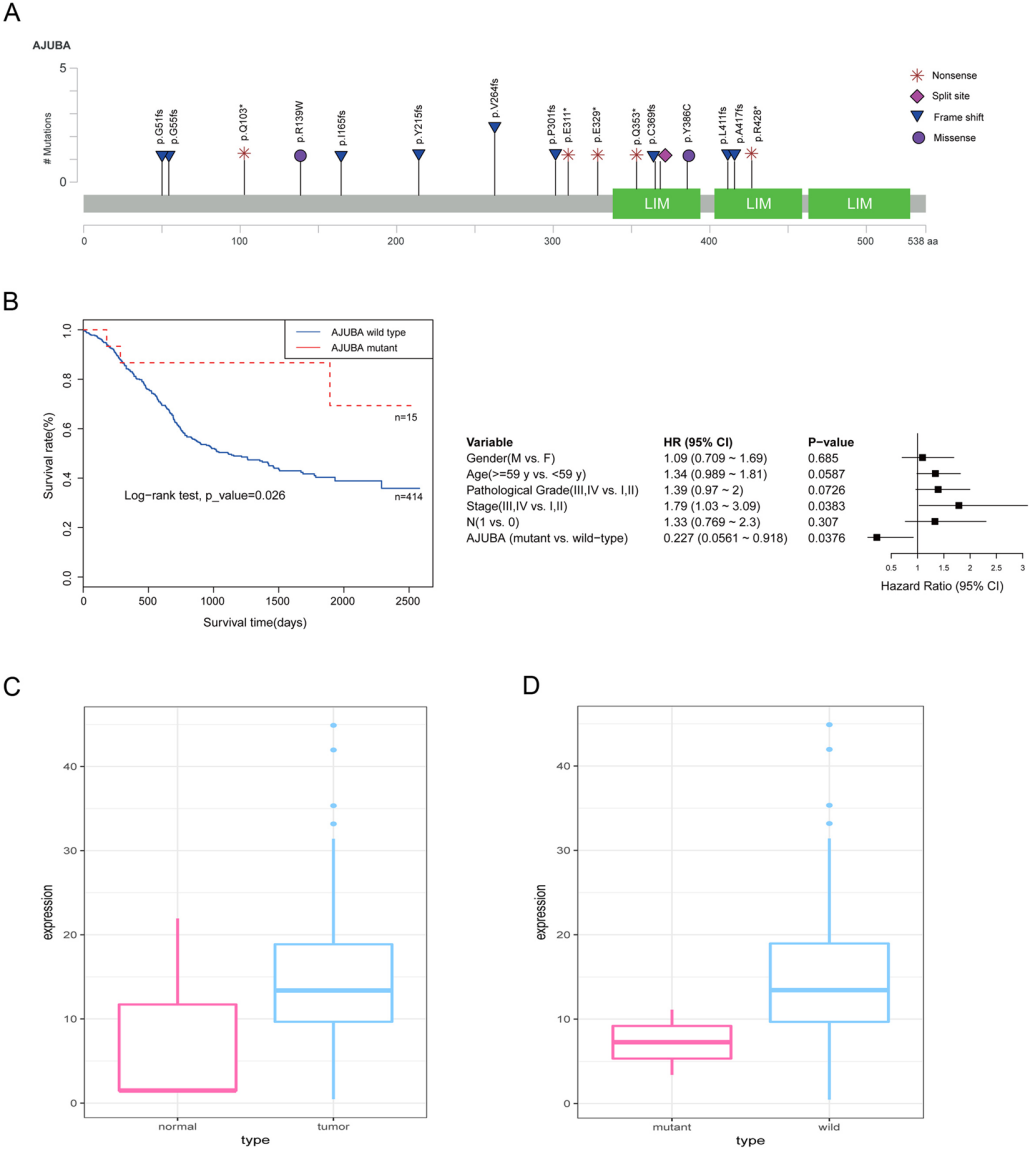
Analysis of AJUBA. (**A**) Somatic mutation types and positions on *AJUBA*. (**B**) Left: Kaplan-Meier survival curve for *AJUBA* was significantly associated with patient survival, *p* < 0.1 was considered statistically significant. Right: Cox proportional hazards model for patients, *p* < 0.05 was considered statistically significant. (**C**) Comparison of the expression of *AJUBA* in tumour and normal samples in the TCGA ESCC cohort. (**D**) Comparison of the expression of *AJUBA* in the *AJUBA* mutant and wild-type samples in the TCGA ESCC cohort.

The MutSigCV method was used to identify SMGs in the 490 ESCC tumours. Finally, 18 SMGs were identified (Fig. 2, Supplementary Table 6), 15 of which had been previously reported in ESCC (*TP53*, *AJUBA*, *CDKN2A*, *KMT2D(MLL2)*, *ZNF750*, *FAT1*, *NOTCH1*, *NOTCH3*, *PIK3CA*, *NFE2L2*, *RB1*, *KDM6A*, *FBXW7*, *CREBBP*, and *TGFBR2*). *CUL3*, *PTEN* and *DCDC1* were identified as novel SMGs in our study. As the most important tumour suppressor, the nonsilent mutation frequency of *TP53* was 84.90% in 490 tumours, which was consistent with previous reports^3,4,7^. In our study, the nonsilent mutation rate of *AJUBA* was 3.9%, including two stop-gain and three frame shift mutations in the LIM domain (Fig. 3A). Survival analysis revealed that *AJUBA* was significantly associated with prognosis (*p* = *0*.*026*, log–rank test, Fig. 3B). We also found the expression level of *AJUBA* was higher in ESCC tumour tissues compared to normal samples, and the expression level of *AJUBA* was lower in *AJUBA*-mutated tumour samples than in the wild-type tumour samples in ESCC cohort (*p* < *0*.*001*, Student’s t-test, Fig. 3C; *p* < *0*.*001*, Student’s t-test, Fig. 3D). *NOTCH1*, which encodes a member of the NOTCH family of proteins, has been reported to be an important gene in many human cancers, including ESCC. We found that *NOTCH1* is significantly correlated with tumour stage (Fisher’s exact test, *p* < 0.001) and lymph node metastasis (Fisher’s exact test, *p* < 0.001), consistent with previous studies^8,15^.

*KDM6A* is another SMG in our study (Supplementary Fig. 2A), which had been reported as a driver gene in ESCC^4^.

We identified six mutations in the phosphatase domain (p.A86T, p.R130*, p.R130Q(2), p.F145I, p.Q171*) and six mutations in the C2 domain (p.G209A, p.F215C, p.K263*, p.Q245*, p.F257S, p.VL317fs) of the tumour repressor *PTEN* (Supplementary Fig. 2A).

We identified 14 somatic mutations in *CUL3* gene, 13 of which were located in the Cullin domain (Supplementary Fig. 2A), which provides a scaffold for ubiquitin ligases (E3).

We identified 4 mutations in the ecTbetaR2 domain of the gene *TGFBR2*, which was also known as transforming growth factor beta receptor 2 ectodomain and transmits signals from the cell surface into the cell. We also identified 7 mutations in another important domain, protein tyrosine kinase, which is a key regulator of normal cellular processes and has a critical role in the development of many cancers^16^.

We identified 15 nonsilent mutations in *DCDC1* gene, of which 11 were missense mutations and 3 were nonsense mutations. We also observed a genomic deletion region containing *DCDC1* in 31 WGS data sets.

### Function classification of SMGs

To further investigate the biological function of cancer-associated genes, we classified SMGs into six categories according to previous functional studies. *TP53*, *CDKN2A*, *NFE2L2*, *RB1*, and *CUL3* were involved in cell cycle and apoptosis regulation (Supplementary Fig. 3A). The histone modifier genes included *KDM6A* and *MLL2*. *AJUBA*, *FAT1*, *FBXW7*, *NOTCH3* and *NOTCH1* were involved in Wnt signalling and the NOTCH pathway. *PIK3CA* and *PTEN* were involved in the PI3 kinase pathway. *PIK3CA* encodes the catalytic subunit of phosphatidyl 3-kinase (PI3K), which is an intracellular central mediator of cell survival signals. *AKT* phosphorylates mTOR (mammalian target of rapamycin), downstream of *PI3K*, and *PTEN* inhibits *AKT* by dephosphorylation. In our study, in addition to finding the nonsense mutations in *PTEN* that could cause loss of function, we also detected hotspot mutations in the p110a domain (p.N345K, p.C420R, p.E545K, p.E542K) and C-terminal portion (p.H1047R, p.H1047L) coded by *PIK3CA*. These hotspot mutations were reported to induce a gain of function in Oncogenicity^17^.

In recent years, frequent mutations in the *NFE2L2/KEAP1/CUL3* pathway had been reported in many types of cancers, including ESCC^10,18–21^. In our study, we identified mutations in *NFE2L2* in 9.6% of the ESCC samples and mutations in *KEAP1* and *CUL3* in 2.9% of the ESCC samples. We found that the mutations in *NFE2L2* were almost mutually exclusive with mutations in *KEAP1* and *CUL3* and that the mutations in *KEAP1* and *CUL3* were mutually exclusive (Supplementary Fig. 2B, Supplementary Table 7).

Histone modification enzymes control the chromatin structure and regulate gene expression^22^. Histone modifications play an important role in the occurrence and development of cancers. We identified two significantly mutated genes (*KMT2D* and *KDM6A*) associated with histone modification. *KMT2D* encoded histone methyltransferase, and promoted the transcriptional activation of target genes through modifying Histone H3 Lysine 4 Trimethylation (H3K4me3)^23^. In our study, the nonsilent mutation frequency of *KMT2D* was 11%, and 30 mutations (46.9%) were truncating (nonsense mutation and frame shift).

### CNA Analysis of ESCC

In the CNA analysis, a total of 57 genomic regions were obtained using 31 WGS data, and 34 focused regions exhibited significant amplification or deletion (*q* < 0.05, Supplementary Table 8, Supplementary Fig. 4), including 11q13.3 amplification and 9p21.3 deletion, which have been reported to be associated with human cancers^3,24^.

We also conducted CNA analysis on 283 WES data, and selected the CNA genes that were recorded in the CGC database for further analysis. CGC is a database that includes genes with substantial published evidence in Oncology. We used this database to select the potentially functional CNA genes.

**Figure 4:**
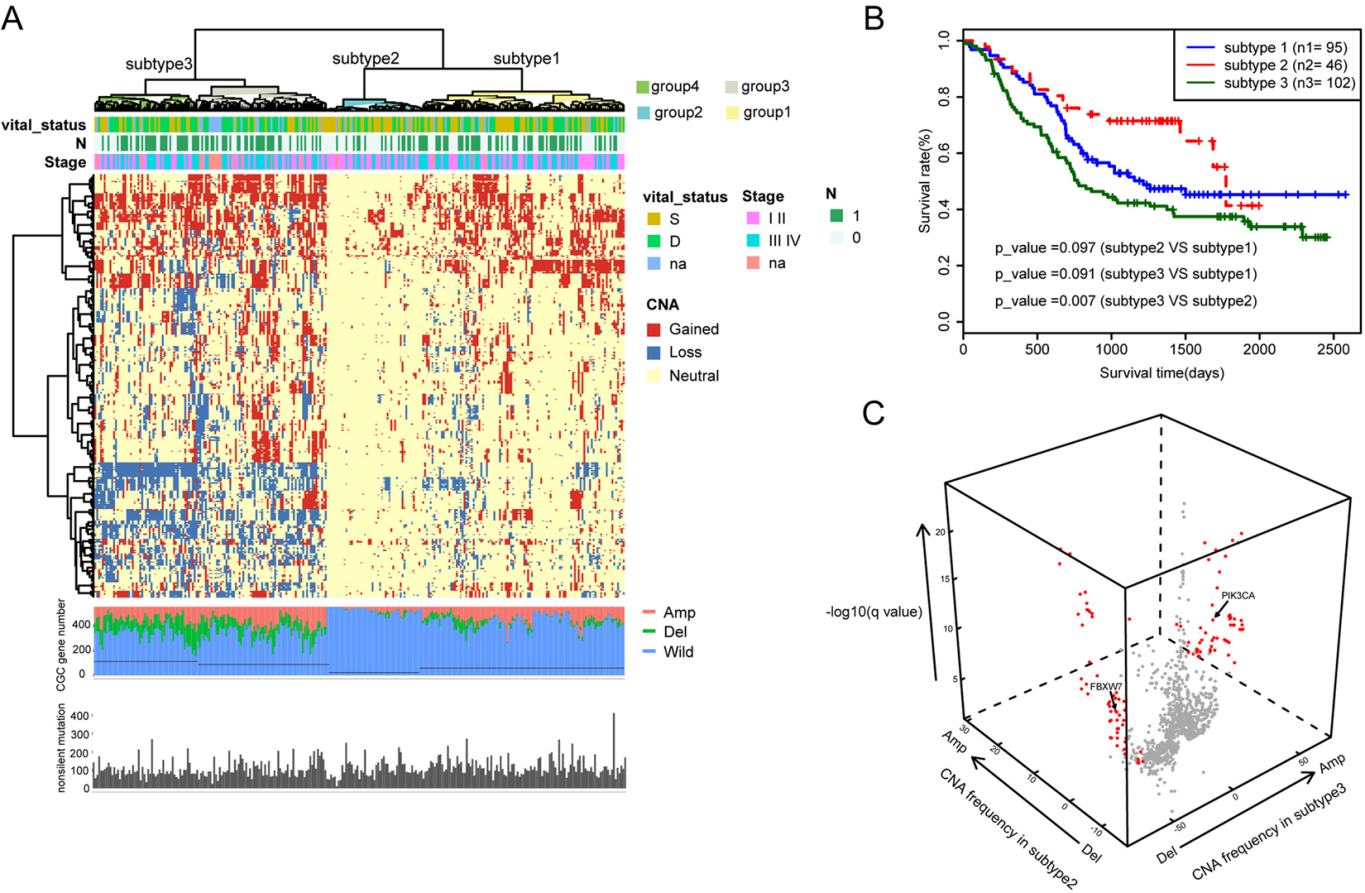
Characterization of ESCC subtypes. (**A**) Hierarchical clustering analysis on the CNA of cancer gene census (CGC). Upper bars: stage, lymphatic metastasis and vital status. Bottom bars: nonsilent mutations of each sample and number of CNA genes. The line represents the median number of CNA genes. (**B**) Kaplan-Meier analysis comparing survival of patients stratified by subtype. (**C**) Multidimensional scaling screen for CGC genes by comparing subtypes 3 and 2. Genes that *q* < 0.001 and frequency of copy number gain or loss in subtypes 3 or 2 >= 40% were highlighted in red.

Hierarchical clustering analysis on the CNA of CGC genes revealed four subgroups within the study patients (Fig. 4A). Group3 and group4 showed a high frequency of CNAs, followed by group1. Patients in group2 exhibited the fewest CNAs and were significantly associated with an early stage (Fisher’s exact test, *p* = *0*.*016*) and fewer lymph node metastases (Fisher’s exact test, *p* = *0*.*005*). Group3 was significantly associated with late stage (Fisher’s exact test, *p* = *0*.*038*). We found that patients in group3 and group4 showed high similarity in the CNA spectrum. Thus, we combined the two groups as subtype3 for additional analysis. Accordingly, group1 and group2 were also renamed subtype1 and subtype2, respectively. By performing Kaplan-Meier analysis, we found that subtype3 marked for worse prognosis compared with patients of subtype2 (*p* = 0.007, Log-rank test, Fig. 4B). Moreover, we performed Student’s t-test to select the most representative CNA genes between subtype3 and subtype2. Finally, 128 genes were identified as significantly differential CNA genes between the two subtypes (*q* < 0.001, gain or loss frequency >= 40% in either subtype, Fig. 4C, Supplementary Table 9).

To further interpret the clinical significances of the representative CNA genes, we annotated the 128 CNA genes with the CIViC database^25^ (Supplementary Table 10). *PIK3CA* amplification was found in 15.7%(8 of 51) and 71.8%(89 of 124) cases in subtype2 and subtype3, respectively, which has been reported to be associated with sensitivity to several drugs in epithelial ovarian cancer, stomach carcinoma and head and neck squamous cell carcinoma^26–28^. *FBXW7* deletion was found in 0%(0 of 51) and 46.8%(58 of 124) cases in subtype2 and subtype3, respectively, and has been reported to be associated with increased sensitivity of drugs in breast cancer and renal cell carcinoma^29,30^.

### Immunogenomic analysis of ESCC

To find immunotherapy clues for ESCC, we comprehensively analysed the immune-related signalling pathways in ESCC. NF-kB is a protein complex that controls cell proliferation and survival and plays an important role in regulating the immune response to infection^31^. In this study, a total of 12 genes, including *TRAF*, *IRAK*, *TAB2*, *TLR*, *IL1R*, and *MYD88*, harboured nonsilent mutations, indicating the activation of the NF-kB signalling pathway in tumour cells. The persistent activation of NF-kB can lead to cell resistance to apoptosis and resistance to chemotherapeutic drug-induced apoptosis. JAK-STAT is a signal transduction pathway that is stimulated by cytokines and is involved in cell proliferation, differentiation, apoptosis, immune regulation and many other important biological processes. JAK-STAT consists of three main components: tyrosine kinase-related receptors, tyrosine kinase JAK and transcription factor STAT^32^. Disrupted JAK-STAT functionality can result in immune deficiency syndromes and cancers^32^. We identified 18 mutated genes in this pathway, 13 of which belong to the three JAK-STAT components.

## Discussion

In this study, we have gathered the published ESCC sequencing data and performed a comprehensive analysis on the largest ESCC cohort currently available. By deciphering the mutational signatures from these 490 ESCC tumours, we identified five mutational signatures. All these five signatures had been reported in ESCC before^7,13,14^. Notably, our survival analysis showed that signature4 was associated with poor survival in ESCC patients (Supplementary Fig. 2C). It was reported that this signature was characterized mainly by C > T mutations and was associated with defective DNA mismatch repair^13^.

To understand the dominant mutational signatures in individual patients, we performed a hierarchical clustering based on the enrichment of specific mutational signatures and identified 3 subgroups of patients. Cluster1 was dominated by signature3, Cluster2 was dominated by signatures 1 and 5, and Cluster3 was dominated by signature2. Notably, we found that Cluster3 indicated a better prognosis compared with Clusters 1 and 2.

Although the causes that may have led to the different survival time among the three ESCC groups remain largely unknown, our results provide an atlas of the molecular subtypes of ESCC with potential prognostic value based on the largest ESCC sample size to date.

Our study also presented a more comprehensive mutational landscape of ESCC. In addition to the well-defined ESCC-related genes, including *TP53*, *AJUBA*, *CDKN2A*, *KMT2D(MLL2)*, *NOTCH1*, *NOTCH3*, *PIK3CA*, *RB1*, *CREBBP*, *NFE2L2*, *ZNF750*, *FAT1*, *KDM6A*, *FBXW7*, and *TGFBR2*, we identified three novel oesophageal cancer-related genes: *PTEN*, *DCDC1* and *CUL3*.

*PTEN* was frequently mutated in other human cancers as an important tumour suppressor^33^, including breast, prostate, gastric cancer and endometrial carcinomas^33–35^. The protein encoded by *PTEN* preferentially dephosphorylates phosphoinositide substrates and inhibits integrin-mediated cell spreading and cell migration^36^. In this study, we identified 4 nonsense mutations in the phosphatase and C2 domain of *PTEN*. These nonsense mutations resulted in truncated proteins, indicating that they may have loss-of-function effects. *CUL3* encodes a member of the cullin protein family, and the encoded protein was reported to form the core component of CUL3-based E3 ligase complex and to play a critical role in the poly-ubiquitination and subsequent degradation of *NFE2L2* protein in lung squamous cell carcinoma^37^. We identified 10 somatic mutations in the Cullin domain that play an essential role in targeting proteins for ubiquitin-mediated degradation^38^. These results indicated that the mutations in *CUL3* gene may affect the degradation of *NFE2L2* protein in ESCC cells. *DCDC1* was another novel SMG identified in our study, which encodes a member of the doublecortin family^39^. We observed frequent nonsilent mutations and deletion this gene, which may cause dysregulated microtubule polymerization and contributes to ESCC development. An important finding here was that the mutations of *AJUBA* were significantly associated with prognosis (*p* = *0*.*026*, log–rank test, Fig. 3B). Most of the mutations identified in *AJUBA* were stop-gain and frame shift mutations that occurred in the LIM domain and were predicted to truncate the protein (Fig. 3A), consistent with a recent report that the expression level of *AJUBA* tended to be lower in *AJUBA*-mutant tumours than in tumours with wild-type *AJUBA* ^4^. However, *AJUBA* was reported to be a binding partner of large tumour suppressor type 2 (LATS2) and to inhibit the proliferation of tumour cells via Hippo signalling cascade^40^. Overexpression of *AJUBA* was also shown to increase the proliferation of head and neck squamous cell carcinoma (HNSCC) cells, and mutations in *AJUBA* were associated with the sensitivity of HNSCC to treatment with cell-cycle inhibitors^41^. More recently, another study showed that the *AJUBA* level was significantly higher in ESCC tissues compared with matched adjacent tissues. *AJUBA* functions as an oncogenic gene in both *in vitro* and *in vivo* experiments^42^.

In this study, we found that loss of functional mutations in *AJUBA* is associated with a better outcome of ESCC patient. This result was consistent with the oncogenic function of *AJUBA* in ESCC and highlighted the potential application of *AJUBA* as a prognostic marker in ESCC.

To further investigate the biological function of cancer-associated genes, we classified the SMGs into different categories according to previous functional studies. Our results showed that the signalling pathways are implicated in ESCC, including cell cycle and apoptosis regulation, histone modification, Wnt pathway, NOTCH pathway, PI3K/AKT pathway, P53 signalling pathway, and Hedgehog signalling pathway. PI3K-AKT is an important signalling pathway that has been identified in human cancers and is involved in regulating cell functions such as proliferation, differentiation, apoptosis, and glucose transport^43^. Inactivation of this pathway was usually caused by mutations in key genes, such as gain-of-function mutations in *PIK3CA* and *AKT*, and loss of function mutations of *PTEN*. The detection of loss of function mutations in *PTEN* and gain of function mutations in *PIK3CA* in our study indicated different mechanisms of dysregulation of the *PIK3CA/AKT* pathway contributing to ESCC development. *NFE2L2* is a transcriptional activator for genes in response to oxidative stress. In tumour cells, mutations of *NFE2L2* were reported to increase resistance to oxidative stress, and promote tumour growth^44^. Notably, we also found that the mutations in *NFE2L2*, *KEAP1*, and *CUL3* were almost mutually exclusive (Supplementary Fig. 3C), which was consistent with the finding in SqCC and HNSCC^19,20^ and indicated that the mutation and dysfunction of the *NFE2L2/KEAP1/CUL3* pathway may contribute to the development of ESCC by increasing the resistance to oxidative stress. Moreover, two significantly mutated genes (*KMT2D* and *KDM6A*) associated with histone modification were identified in our study. These findings have deepened our understanding on the molecular mechanisms underlying the tumourigenesis of ESCC.

Through immunogenomic analysis, we detected several key gene mutations in the immune pathway, and the effects of these mutations on the immune mechanisms need to be further studied.

By analysing potentially functional CNA genes (according to CGC) from the WES data and performing hierarchical clustering analysis of these genes, we identified 4 ESCC subgroups with different CNA gene numbers and clinical relevance (Fig. 4). Notably, patients in group2 exhibited fewer CNAs and were significantly associated with early stages and fewer lymph node metastases. Group3 exhibited higher CNAs and was significantly associated with late stages. These results suggest the links between the CNA background and tumour progression of ESCC and provide a novel genomic classification method that may help differentiate ESCC patients with dissimilar tumour stages and metastasis statuses. As a result, different therapy strategies could be chosen accordingly.

Moreover, Kaplan-Meier analysis showed that the combination of group3 and group4 (subtype3) marked for worse patient prognosis compared with patients of group2 (subtype2). This result indicates that higher CNAs in the CGC genes are associated with poor patient prognosis and suggest the potential utility of CNA data of these CGC genes as prognostic marker in ESCC.

Given the differences of clinical features between subtype2 and subtype3, it is intriguing to identify the representative CNA genes between these two subtypes, and to find potential therapeutic target for the patients in different subtypes. By comparing the CNA values in subtype3 and subtype2 and annotated the CNA genes with the CIViC database, we identified two high frequency CNA genes which had been associated with drug responses in different cancers by the former studies. Notably, high frequency amplification of *PIK3CA* gene was found in subtype3 (71.8%), however the amplification frequency of this gene in subtype2 was low (15.7%). And according to the annotation result of CIViC, we found that *PIK3CA* amplification was associated with partial response to treatment with PI3K inhibitor pictilisib (GDC-0941) in epithelial ovarian cancer patients^26^, and positively associated with the sensitive of PI3K inhibitor in stomach carcinoma and head and neck squamous cell carcinoma (HNSCC)^27,28^. Given that the CNA spectrums was similar between ESCC and HNSCC^3^, our results suggest that *PIK3CA* amplification may also serve as a therapeutic target for PI3K inhibitor in ESCC. *FBXW7* is another high frequency CNA gene associated with drug responses. We identified deletion of *FBXW7* gene in 58/124 (46.8%) of patients in subtype3, but found no deletion of this gene in subtype2. And according to the annotation result of CIViC, we found that *FBXW7* deletion enhanced the sensitivity to mTOR inhibitors in breast cancer and renal cell carcinoma^29,30^.

Although further clinical trials are still needed, the dramatically differences of *FBXW7* deletion and *PIK3CA* amplification between the subtype2 and subtype3 in our ESCC cohort, suggested that mTOR inhibitors and PI3K inhibitors may also suit for certain groups of ESCC patient. And the molecular subtyping base on CNA data may be helpful to classify ESCC patients for different drugs. These analyses have shed some light into the potential application of the CNAs as therapeutic target in ESCC.

To sum up, we have performed a comprehensive genomic analysis on the largest ESCC cohort. We identified SMGs, mutational signatures, and subtypes of ESCC related to prognosis. We also identified potential therapeutic targets for special subtype of ESCC. Our analysis deepened our understanding of the heterogeneity of ESCC and shed light on the molecular mechanisms and pathways underlying ESCC. These studies may provide a potential improvement to the strategies that are used for the therapy and prognosis of ESCC patients.

## Methods

### Genome data collection and processing

We collected fastq or somatic mutations data of 492 paired ESCC samples from seven publications (Supplementary Table 1), consisting of 41 whole-genome sequences and 451 whole-exome sequences. The clinical data were also acquired (Supplementary Table 2). Of the 492 cases of ESCC, the fastq data of 323 cases from 4 publications^3,4,6,7^ were re-analysed with our standard pipeline, and somatic mutations from the remaining publications were combined for further analysis. To improve the accuracy and comparability of the data, we eliminated one hyper-mutant sample and filtered the false positive mutations with our own panel of normal datasets and the Exome Aggregation Consortium (ExAC) database. Finally, 490 cases were used for further analysis.

The Fastq data from 323 cases were processed according to the following pipeline. Low-quality reads with more than five unknown bases and sequencing adaptors were removed. The remaining high-quality reads were aligned to NCBI human reference (hg19) using BWA^45^. Picard (http://broadinstitute.github.io/picard/) was used to mark duplicates, and Genome Analysis Toolkit^46^ (v.1.0.6076, GATK IndelRealigner) to improve the accuracy of the genome alignment. Somatic point mutations were detected using muTect^47^. Somatic Indels were detected with GATK Somatic Indel Detector. The somatic variations combined the remaining publications’ somatic mutations were annotated with Oncotator^48^.

To further enhance the accuracy of somatic mutations, we filtered the false positive mutations with a threshold of greater than 5% of mutation frequency in normal samples according to our panel of normal bams, and a threshold of greater than 1% in the Exome Aggregation Consortium (ExAC) database.

Copy number alterations (CNAs) were first detected with SegSeq for 31 WGS, and GATK4 Alpha for 283 WES. GISTIC2.0^49^ was performed to identify significantly amplified or deleted genomic regions. Hierarchical clustering was used to identify sample subtypes. Student’s two-sided t-test was used to select significant differential CNAs between subtype3 and subtype2. *P* values were adjusted using the R package ‘p.adjust’, and *q* < 0.001 was defined as statistically significant. We employed CIViC^25^ to identify CNA genes associated response a targeted therapy.

### Mutational signature analysis

The mutational signatures were displayed using a 96-context classification. A nonnegative matrix factorization (NMF) was used to identify the operative processes based upon the reproducibility of the signatures and low error for reconstructing the original catalogues.

### Identification of significantly mutated genes

Significantly mutated genes (SMGs) were detected using MuSigCV (mutation significance with covariates), which identifies genes highly relevant to cancer rather than high frequency mutations by considering the background mutation events. A gene was considered to be a SMG if it satisfied the condition for statistical significance (*q < 0*.*1*) at MuSigCV.

### Analysis of clinical pathological data

The survival rate was calculated by the Kaplan-Meier method, and the difference was compared by the Log-rank method. Cox proportional hazards model was used for the analysis of hazards, as implemented in the R package ‘survival’ (http://cran.r-project.org/web/ packages/survival/). We removed the patients whose survival information were unavailable. By univariate analyses, the significance of the clinical variables was *p* < *0*.*1* level. Multivariate analyses were performed using age, gender, pathological grade, N, and stage as covariates, and the significance of clinical multivariates was *p* < *0*.*05*.

## Electronic supplementary material

Supplementary information

Dataset 1

Dataset 2

Dataset 3

Dataset 4

Dataset 5

Dataset 6

Dataset 7

Dataset 8

Dataset 9

Dataset 10

Dataset 11

Dataset 12

Dataset 13

## References

[1] DeSantis CE (2014). Cancer treatment and survivorship statistics, 2014. CA Cancer J. Clin..

[2] Chen W (2016). Cancer statistics in China, 2015. CA Cancer J. Clin..

[3] Song Y (2014). Identification of genomic alterations in oesophageal squamous cell cancer. Nature.

[4] Gao YB (2014). Genetic landscape of esophageal squamous cell carcinoma. Nat. Genet..

[5] Lawrence MS (2014). Discovery and saturation analysis of cancer genes across 21 tumour types. Nature.

[6] Lin DC (2014). Genomic and molecular characterization of esophageal squamous cell carcinoma. Nat. Genet..

[7] Zhang L (2015). Genomic analyses reveal mutational signatures and frequently altered genes in esophageal squamous cell carcinoma. Am. J. Hum. Genet..

[8] Qin HD (2016). Genomic characterization of esophageal squamous cell carcinoma reveals critical genes underlying tumorigenesis and poor prognosis. Am. J. Hum. Genet..

[9] Agrawal N (2012). Comparative genomic analysis of esophageal adenocarcinoma and squamous cell carcinoma. Cancer Discov..

[10] Cancer Genome Atlas Research, N. *et al* Integrated genomic characterization of oesophageal carcinoma. *Nature* **541**, 169-175, doi:10.1038/nature20805 (2017) 10.1038/nature20805 PMC5651175 28052061

[11] Harris RS, Petersen-Mahrt SK, Neuberger MS (2002). RNA editing enzyme APOBEC1 and some of its homologs can act as DNA mutators. Mol. Cell.

[12] Caval V, Suspene R, Vartanian JP, Wain-Hobson S (2014). Orthologous mammalian APOBEC3A cytidine deaminases hypermutate nuclear DNA. Mol. Biol. Evol..

[13] Rosenthal R, McGranahan N, Herrero J, Taylor BS, Swanton C (2016). DeconstructSigs: delineating mutational processes in single tumors distinguishes DNA repair deficiencies and patterns of carcinoma evolution. Genome Biol..

[14] Alexandrov LB (2013). Signatures of mutational processes in human cancer. Nature.

[15] Cheng C (2016). Genomic analyses reveal FAM84B and the NOTCH pathway are associated with the progression of esophageal squamous cell carcinoma. GigaScience.

[16] Zwick E, Ullrich BJ (2001). A. Receptor tyrosine kinase signalling as a target for cancer intervention strategies. Endocr Relat Cancer.

[17] Gymnopoulos M, Elsliger MA, Vogt PK (2007). Rare cancer-specific mutations in PIK3CA show gain of function. Proc. Natl. Acad. Sci. USA.

[18] Sawada G (2016). Genomic Landscape of Esophageal Squamous Cell Carcinoma in a Japanese Population. Gastroenterology.

[19] Cancer Genome Atlas Research, N. (2012). Comprehensive genomic characterization of squamous cell lung cancers. Nature.

[20] Cancer Genome Atlas, N. (2015). Comprehensive genomic characterization of head and neck squamous cell carcinomas. Nature.

[21] Cancer Genome Atlas Research, N. (2014). Comprehensive molecular characterization of urothelial bladder carcinoma. Nature.

[22] Bannister AJ, Kouzarides T (2011). Regulation of chromatin by histone modifications. Cell Res..

[23] Kerimoglu C (2013). Histone-methyltransferase MLL2 (KMT2B) is required for memory formation in mice. J. Neurosci..

[24] Secrier M (2016). Mutational signatures in esophageal adenocarcinoma define etiologically distinct subgroups with therapeutic relevance. Nat. Genet..

[25] Griffith M (2017). CIViC is a community knowledgebase for expert crowdsourcing the clinical interpretation of variants in cancer. Nature genetics.

[26] Sarker D (2015). First-in-human phase I study of pictilisib (GDC-0941), a potent pan–class I phosphatidylinositol-3-kinase (PI3K) inhibitor, in patients with advanced solid tumors. Clinical cancer research.

[27] Fritsch C (2014). Characterization of the novel and specific PI3Kα inhibitor NVP-BYL719 and development of the patient stratification strategy for clinical trials. Molecular cancer therapeutics.

[28] Zumsteg ZS (2016). Taselisib (GDC-0032), a potent β-sparing small molecule inhibitor of PI3K, radiosensitizes head and neck squamous carcinomas containing activating PIK3CA alterations. Clinical Cancer Research.

[29] Okazaki H (2014). Circadian regulation of mTOR by the ubiquitin pathway in renal cell carcinoma. Cancer research.

[30] Mao J-H (2008). FBXW7 targets mTOR for degradation and cooperates with PTEN in tumor suppression. Science.

[31] Smith EM, Gregg M, Hashemi F, Schott L, Hughes TK (2006). Corticotropin releasing factor (CRF) activation of NF-kappaB-directed transcription in leukocytes. Cell Mol. Neurobiol..

[32] Aaronson DS, Horvath CM (2002). A road map for those who don’t know JAK-STAT. Science.

[33] Yang Z (2013). Reduced expression of PTEN and increased PTEN phosphorylation at residue Ser380 in gastric cancer tissues: a novel mechanism of PTEN inactivation. Clin. Res. Hepatol. Gastroenterol..

[34] Kong D (1997). PTEN1 is frequently mutated in primary endometrial carcinomas. Nat. Genet..

[35] Li J (1997). PTEN, a putative protein tyrosine phosphatase gene mutated in human brain, breast, and prostate cancer. Science.

[36] Gu J, Tamura M, Yamada KM (1998). Tumor suppressor PTEN inhibits integrin- and growth factor-mediated mitogen-activated protein (MAP) kinase signaling pathways. J. Cell Biol..

[37] Zhang Y (2016). Mutations and expression of the NFE2L2/KEAP1/CUL3 pathway in Chinese patients with lung squamous cell carcinoma. J. Thorac. Dis..

[38] Bosu DR, Kipreos ET (2008). Cullin-RING ubiquitin ligases: global regulation and activation cycles. Cell Div..

[39] Zeng L (2003). Identification of a novel human doublecortin-domain-containing gene (DCDC1) expressed mainly in testis. J. Hum. Genet..

[40] Tanaka I (2015). LIM-domain protein AJUBA suppresses malignant mesothelioma cell proliferation via Hippo signaling cascade. Oncogene.

[41] Zhang M (2017). Mutations of the LIM protein AJUBA mediate sensitivity of head and neck squamous cell carcinoma to treatment with cell-cycle inhibitors. Cancer Lett..

[42] Shi X (2016). AJUBA promotes the migration and invasion of esophageal squamous cell carcinoma cells through upregulation of MMP10 and MMP13 expression. Oncotarget.

[43] Morgan TM, Koreckij TD, Corey E (2009). Targeted therapy for advanced prostate cancer: inhibition of the PI3K/Akt/mTOR pathway. Curr. Cancer Drug Targets.

[44] Hayes JD, McMahon M (2009). NRF2 and KEAP1 mutations: permanent activation of an adaptive response in cancer. Trends Biochem. Sci..

[45] Li H, Durbin R (2009). Fast and accurate short read alignment with Burrows-Wheeler transform. Bioinformatics.

[46] McKenna A (2010). The genome analysis toolkit: a MapReduce framework for analyzing next-generation DNA sequencing data. Genome Res..

[47] Cibulskis K (2013). Sensitive detection of somatic point mutations in impure and heterogeneous cancer samples. Nat. Biotechnol..

[48] Ramos AH (2015). Oncotator: cancer variant annotation tool. Hum. Mutat..

[49] Mermel CH (2011). GISTIC2. 0 facilitates sensitive and confident localization of the targets of focal somatic copy-number alteration in human cancers. Genome biology.

