# Supplementary material for: Comprehensive genomic analysis of Oesophageal Squamous Cell Carcinoma reveals clinical relevance
Source: Sci Rep. 2017 Nov 10;7:15324. doi: 10.1038/s41598-017-14909-5 (PMC5681595; doi:10.1038/s41598-017-14909-5)
Supplement: Supplementary file 1 — Supplementary information [file 41598_2017_14909_MOESM1_ESM.pdf]

Comprehensive genomic analysis of Oesophageal Squamous Cell Carcinoma reveals clinical relevance

Peina Du[1,*], Peide Huang[1,2,*], Xuanlin Huang[1,*], Xiangchun Li[1,3], Zhimin Feng[1], Fengyu Li[1], Shaoguang Liang[1], Yongmei Song[4], Jan Stenvang[2], Nils Brünner[2], Huanming Yang[1,5], Yunwei Ou[6], Qiang Gao[1] & Lin Li[1,7]

[1]BGI-Shenzhen, Shenzhen 518083, China.

[2]Section of Molecular Disease Biology, Department of Drug Design and Pharmacology, Faculty of Health and Medical Sciences, University of Copenhagen, 2200 Copenhagen N, Denmark

[3]Department of Epidemiology and Biostatistics, Tianjin Medical University Cancer Institute and Hospital, Tianjin 300060, People's Republic of China.

[4]State Key Laboratory of Molecular Oncology, Cancer Institute and Cancer Hospital, Chinese Academy of Medical Sciences and Peking Union Medical College, Beijing 100021, China.

[5]James D. Watson Institute of Genome Sciences, Hangzhou, China.

[6]Department of neurosurgery, Beijing tiantan hospital, capital medical university, Beijing 100050, China.

[7]Shanghai Clinical Center for Endocrine and Metabolic Diseases, Shanghai Key Laboratory for Endocrine Tumours, Rui-Jin Hospital, Shanghai Jiao-Tong University School of Medicine, Shanghai, 200025, China.

[*]These authors contributed equally to this work.

Correspondence should be addressed to L.L. (lilin@bgi.com) or Q.G.(gaoqiang@bgi.com)

Supplementary Figures

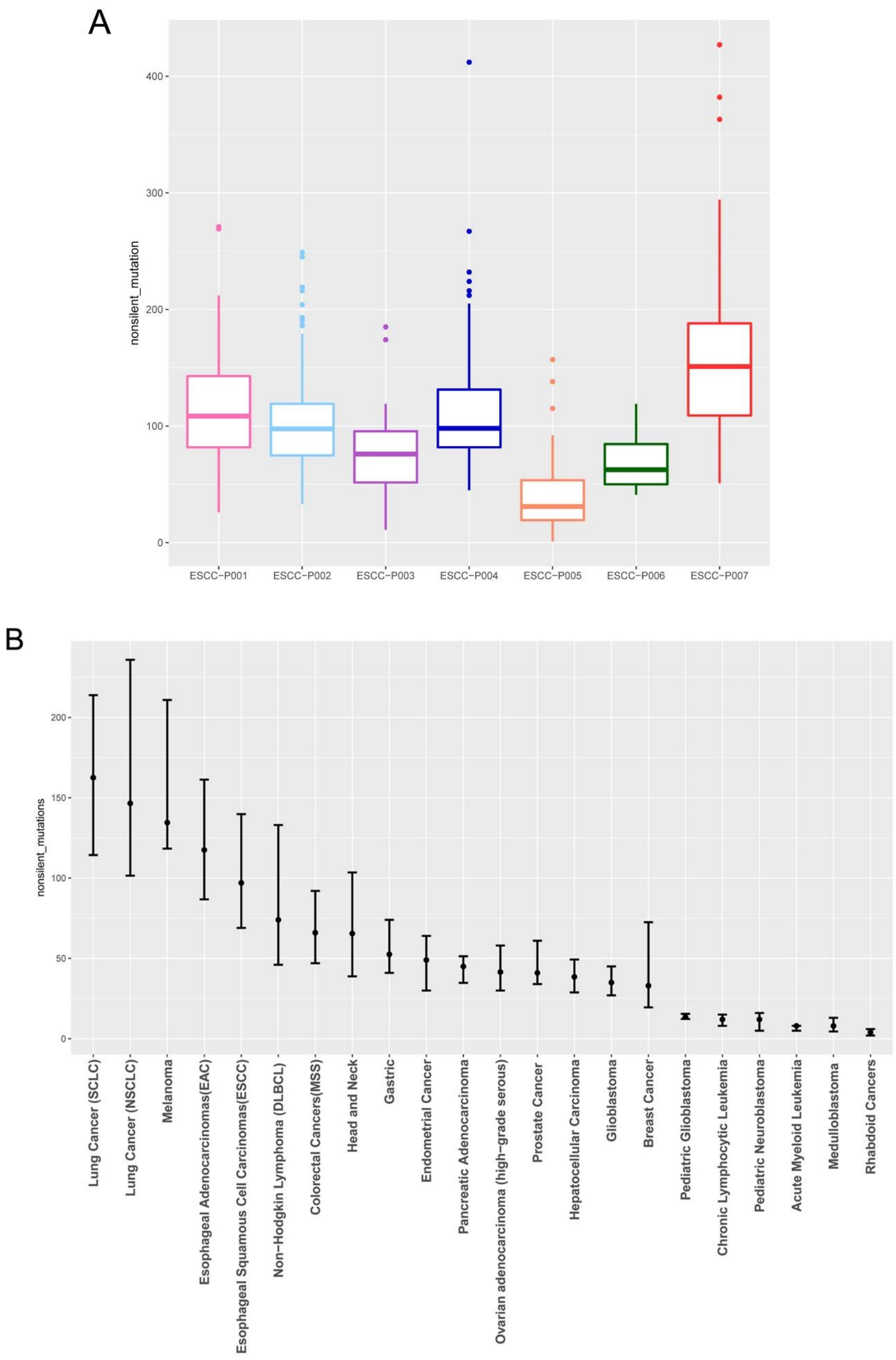

Supplementary Figure 1: Nonsilent mutations per tumour across cancer types. (A) Distribution of nonsilent mutations per tumour in each publication. (B) comparison of nonsilent mutations between cancer types. Horizontal bars indicate the 25 and 75% quartiles.

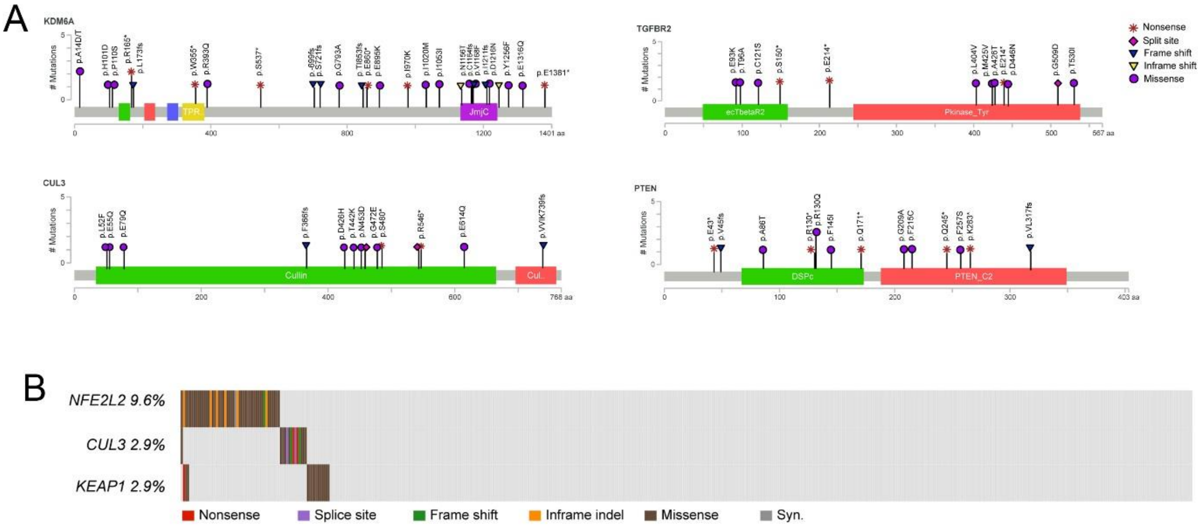

Supplementary Figure 2: (A) Somatic mutation types and positions on *KDM6A*, *CUL3*, *PTEN* and *TGFBR2*. (B) *NFE2L2/KEAP1/CUL3* mutations in ESCC cohort.

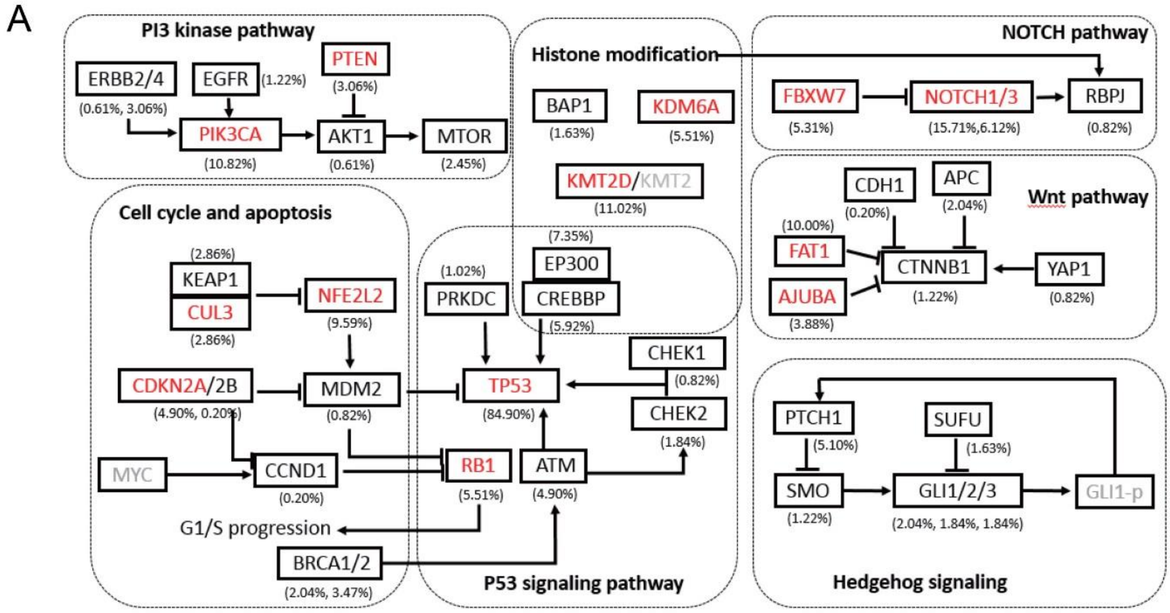

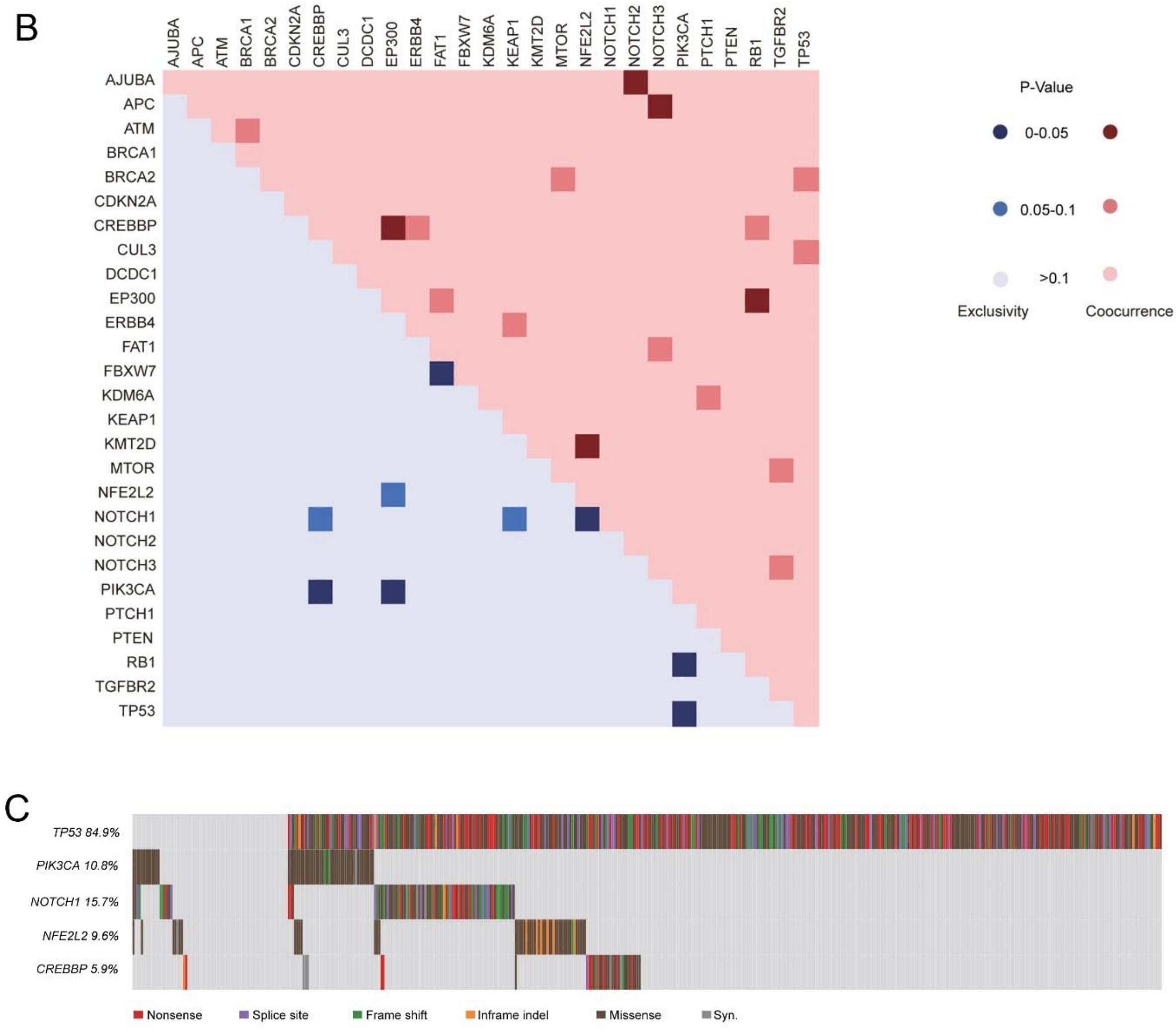

Supplementary Figure 3: Cancer associated pathways altered in ESCC. (A) ESCC mutant genes and their mutation frequencies were pointed to the following important pathways: PI3 kinase pathway, Cell cycle and apoptosis, Histone modification, P53 signaling, hedgehog signaling, Wnt pathway and NOTCH pathway. Significantly mutated genes identified by MutSigCV were colored in red, genes that have no mutations were marked in gray. (B) Permutation analysis of ESCC related pathway genes. (C) *TP53/PIK3CA/NOTCH1/NFE2L2/CREBBP* mutations in ESCC cohort.

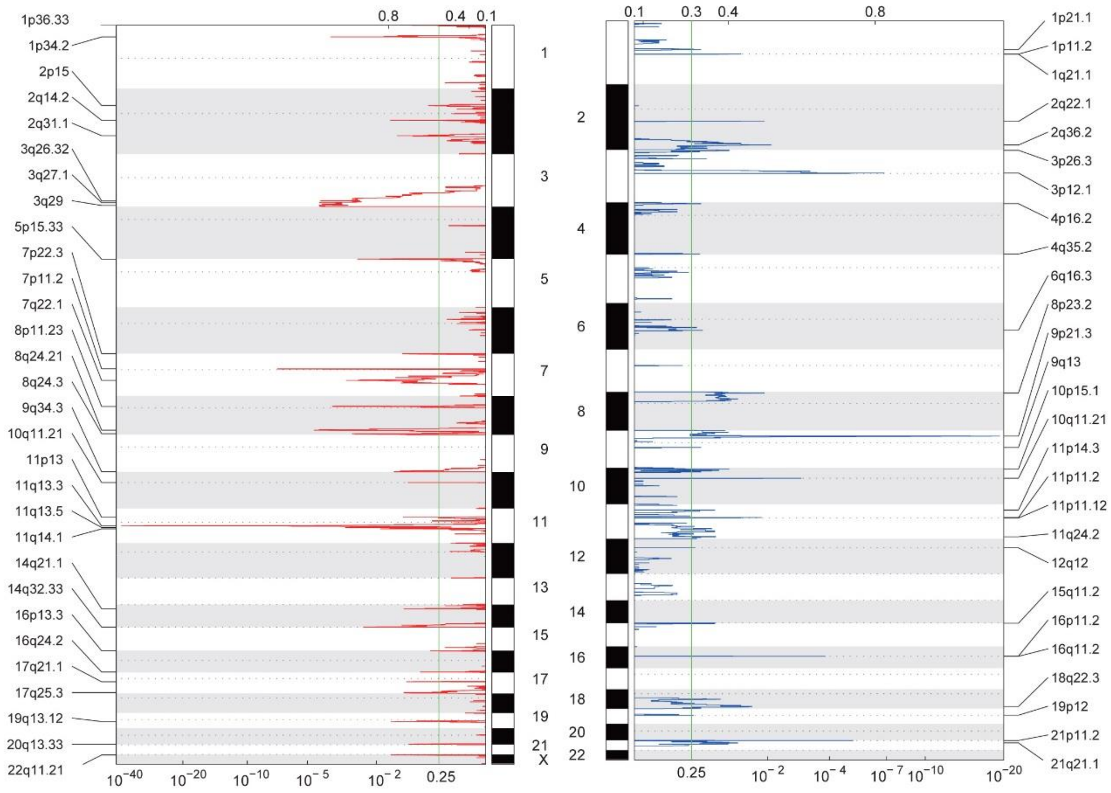

Supplementary Figure 4: Genomic focal regions of CNAs detected in 31 WGS data using GISTIC2.0. Left: GISTIC amplification. Right: GISTIC deletion.

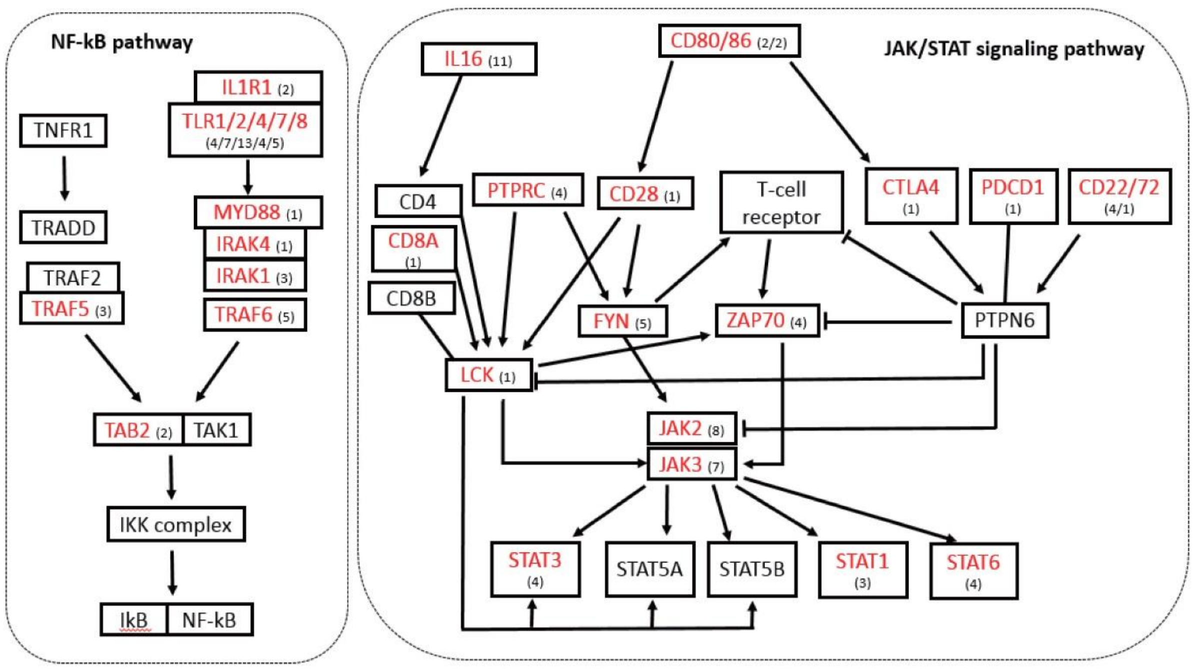

Supplementary Figure 5: Immune-related pathways altered in ESCC. mutated genes identified were colored in red. Numeric represents the number of mutant samples.

Supplementary Tables:

Supplementary Table 1. Genome-wide sequencing studies of ESCC

Supplementary Table 2. Clinical informations of ESCCs

Supplementary Table 3. Somatic mutations in ESCC

Supplementary Table 4. Comparison of nonsilent mutations between ESCC and other cancer types

Supplementary Table 5. Mutation spectrum and signature of ESCC

Supplementary Table 6. Significant Mutated Genes of ESCC

Supplementary Table 7. Permutation analysis of high frequency pathway genes in ESCC

Supplementary Table 8. Significant peaks performed by GISTIC2.0 in 31 WGS

Supplementary Table 9. Significant differential CGCs between subtypes 3 and 2 ($q<0.001$) in 283 WES CNA analysis

Supplementary Table 10. Potential drugs of significant differential CGCs between subtypes 3 and 2

Supplementary Table 11. Mutated genes in ESCC

Supplementary Table 12. Mutation type of ESCC

Supplementary Table 13. Significant Mutated Genes distribution across ESCC projects
